# Physician Preparedness in Response to the Coronavirus Disease 2019 Pandemic: A Cross-Sectional Study From a Developing Country

**DOI:** 10.7759/cureus.10383

**Published:** 2020-09-11

**Authors:** Laila Hashim, Hamza R Khan, Irfan Ullah, Maida Khalid, Talal Almas, Syed Muhammad Jawad Zaidi, Maryam Ehtesham, Muhammad Ali Niaz, Absam Akbar, Abdul Haadi

**Affiliations:** 1 Internal Medicine, Fatima Jinnah Medical University, Lahore, PAK; 2 Internal Medicine, Quaid-e-Azam Medical College, Bahawalpur, PAK; 3 Internal Medicine, Kabir Medical College, Peshawar, PAK; 4 Internal Medicine, Naseer Teaching Hospital, Peshawar, PAK; 5 Internal Medicine, Foundation University Medical College, Islamabad, PAK; 6 Internal Medicine, Royal College of Surgeons in Ireland, Dublin, IRL; 7 Internal Medicine, Rawalpindi Medical University, Rawalpindi, PAK; 8 Surgery, Royal College of Surgeons in Ireland, Dublin, IRL; 9 Internal Medicine, Aga Khan University, Karachi, PAK; 10 Internal Medicine, Royal College of Surgeons In Ireland, Dublin, IRL

**Keywords:** preparedness, coronavirus disease 2019 (covid-19), doctors

## Abstract

Background

December 2019 marked the inception of a global pandemic, with cases being reported worldwide. In the developing nations with scarce healthcare resources, the reliance on healthcare workers who are amply prepared to withstand the prevailing scenario is indispensable. Our study aimed to assess the level of preparedness of doctors working in various hospitals across Pakistan to combat coronavirus disease 2019 (COVID-19).

Methods

We conducted an online questionnaire-based survey in May 2020 to estimate the level of preparedness of doctors working in various departments of various private and public hospitals across Pakistan. The survey comprised 36 questions, with items evaluating the provision of adequate protective equipment, training, mental health resources, and sound collaboration between healthcare workers and the hospital management during the COVID-19 crisis.

Results

A total of 346 doctors responded to the survey, among whom 56.4% were working in public sector hospitals and 46.5% were working more than five days per week. Of those included, 87.6% were being provided with disposable gloves, but 72.8% and 43.4% of respondents professed to having no access to eye protective equipment and gowns, respectively. Only 35.3% of respondents claimed to be trained regarding the use of personal protective equipment and 28.95% were being tested. Of the physicians, 43.4% claimed to have no proper triage system for the suspected patients and 98.3% were concerned about transmitting the disease to their family members. Of the doctors, 53.5% reported that there was sound collaboration between the hospital management and healthcare staff.

Conclusion

The survey provided evidence of inadequate delivery of personal protective equipment and training to doctors working in various hospitals across Pakistan. A sound collaboration between the hospital management and departments needs to be addressed.

## Introduction

In December 2019, patients presenting with respiratory symptoms of unknown etiology were being identified in Wuhan city, Hubei province, China. The infectious agent causing unusual respiratory symptoms was identified to be a novel coronavirus or severe acute respiratory syndrome coronavirus (SARS-CoV-2) by the Chinese health authority while the world health organization (WHO) named the disease caused by this virus as coronavirus disease 2019 (COVID-19) [1]. On March 11, 2020, the WHO categorized COVID-19 as a pandemic [2].

The infectious agent responsible for causing COVID-19 is transmitted via respiratory droplets from infected individuals and remains viable on non-living objects under appropriate atmospheric conditions for several days [3]. The symptoms of COVID-19 may range from a mild to a severe presentation, causing acute respiratory distress syndrome (ARDS), thereby requiring mechanical ventilation [4]. The virus was confirmed to have reached Pakistan on 26 February 2020, and by 28 March, cases were registered in all four provinces of Pakistan [5].

Infected individuals can transmit the source of infection to medical professionals who are particularly prone to contracting infectious diseases even from asymptomatic patients [6]. Due to the human-to-human transmission of COVID-19, the disease is a serious threat to them, especially in developing nations like Pakistan with limited healthcare resources [1,3]. Healthcare workers have the responsibility of not only treating the infected patients but also of acting appropriately to manage the suspected cases and limit spread. Due to the increasing burden of cases and threats to their health, healthcare workers may be subjected to a certain level of psychological distress.

In the prevailing circumstances, the adequate delivery of protective equipment, high level of training, proper communication, and delivery of mental health facilities to the frontline doctors is of utmost importance. Our survey aimed to assess this level of preparedness among the doctors working at various departments of the hospitals in Pakistan to manage COVID-19. To the best of our knowledge, this is the first study to address the gap in knowledge regarding the level of preparedness of our frontline healthcare workers. This study can serve as important evidence to promote the delivery of protective equipment, adequate training, and mental health facilities among doctors.

## Materials and methods

A descriptive cross-sectional study was conducted during May 2020 using an online questionnaire made on Google Forms. A total of 346 doctors and post-graduate residents currently working in the various hospitals in public and private sectors across Pakistan responded to the survey. The questionnaire comprised of 36 questions, including questions regarding the demographics of study participants, the provision of personal protective equipment (PPE) and training, department/hospital preparation in dealing with COVID-19, the practice of doctors in combatting COVID-19, and the perception of doctors regarding COVID-19. The responses of the doctors to various questions were tabulated and their association across the public and private sectors was assessed using the Chi-square test. The data were eventually analyzed using the Statistical Package for the Social Sciences (SPSS) version 25 (IBM Corp., Armonk, NY).

## Results

A total of 346 participants responded to the online survey. The mean age of the participants was 28.88 ± 6.246 years. The demographics of the study participants are delineated in Table 1.

**Table 1:** Demographics of study participants SARS-CoV-2: severe acute respiratory syndrome coronavirus 2

Parameters		Frequency	Percentages
Gender	Male	161	46.5%
	Female	185	53.5%
Marital status	Unmarried	227	65.6%
	Married	119	34.4%
System	Private	151	43.6%
	Government	195	56.4%
Hospital category	Specialized tertiary care teaching hospitals	271	78.3%
	District/Tehsil headquarter hospitals	12	3.5%
	Rural healthcare units	4	1.2%
	Private clinics	10	2.9%
	Others	49	14.1%
Job designation	House officer	118	34.1%
	Medical officer	39	11.3%
	Postgraduate trainee	138	39.9%
	Consultant	34	9.8%
	Other	17	4.9%
Specialty	Cardiology	121	35%
	General Surgery	66	19.1%
	Internal Medicine	46	13.3%
	Gynecology	23	6.6%
	Pediatrics	19	5.5%
	Emergency	13	3.8%
	Other	58	16.8%
How many days of a week do you work?	< 3 days	72	20.8%
	3 to 5 days	113	32.7%
	> 5 days	161	46.5%
Is your department providing only emergency services to the patients in the recent COVID-19 crisis?	No	148	42.8%
	Yes	198	57.2%
Exposure to SARS-CoV-2	Rarely	63	18.2%
	Occasionally	176	50.9%
	Daily	100	28.9%
	Never	07	2.0%

Furthermore, results obtained from inquiring about the provision of personal protective equipment, including disposable gloves and isolation gowns, and training pertaining to protective measures, methods of nasopharyngeal swab testing, and how to use and dispose of personal protective equipment, are elucidated in Table 2. 

**Table 2:** Provision of PPE and training PPE: personal protective equipment; COVID-19: coronavirus disease 2019 The data are expressed in the form of frequency (percentages).

Questions	Yes	No
Questions regarding the provision of PPE		
Have you been provided with disposable gloves in your department?	303 (87.6%)	43 (12.4%)
Have you been provided with an isolation gown in your department?	196 (56.6%)	150 (43.4%)
Have you been provided with eye-protective goggles in your department?	94 (27.2%)	252 (72.8%)
Have you been provided with alcohol-based sanitizers in your department?	281 (81.2%)	65 (18.8%)
Questions regarding the provision of training		
Have you been given training regarding protective measures to be taken while encountering a patient suspected of having COVID-19?	122 (35.3%)	224 (64.7%)
Have you been introduced to the methods of nasopharyngeal/oropharyngeal swab sampling?	100 (28.9%)	246 (71.1%)
Have you been given training on how to use and dispose of personal protective equipment?	110 (31.8%)	236 (68.2%)

The preparation levels of the various hospitals in response to COVID-19 is further tabulated in Table 3. 

**Table 3:** Department/hospital preparation for COVID-19 COVID-19: coronavirus disease 2019 Data is expressed in frequency (percentages).

Questions	Yes	No
Is there a proper triage system available in your department to receive and screen patients suspected for COVID-19?	196 (56.6%)	150 (43.4%)
Is portable imaging equipment available in your department to limit the transport of COVID-19 suspected or confirmed patients?	196 (56.6%)	150 (43.4%)
Do you have mental health and counseling resources in your department for health care workers?	51 (14.7%)	295 (85.3%)
Is there sound collaboration between healthcare representatives of your department and hospital management?	185 (53.5%)	161 (46.5%)

Additionally, the physicians were inquired about their adaptations towards combatting the COVID-19 pandemic. In specific, they were asked about the use of an N95 mask, how frequently they change their mask, how they disinfect their equipment, and if they notify their hospital's infection prevention team or local health department when a COVID-19 suspected patient is being investigated (Table 4).

**Table 4:** Physician adaptation in response to COVID-19. COVID-19: coronavirus disease 2019

Parameters	Frequency	Percentages
Do you use an N-95 mask or higher when in close contact with a patient under investigation for COVID-19?		
No	98	28.3%
Yes	248	71.7%
How frequently do you change the protective mask you use?		
After contact with every patient	9	2.6%
Daily	114	32.9%
Every alternate day	63	18.2%
Weekly	111	32.1%
Never	49	14.2%
Do you disinfect your equipment (stethoscope, manometer) after contact with every COVID-19 suspected patient?		
No	63	18.2%
Yes	283	81.8%
Do you immediately notify your hospital's infection prevention team or local health department, when a COVID-19 suspected patient is being investigated?		
No	46	13.3%
Yes	300	86.7%

Table 5 elucidates the physicians' perception regarding the COVID-19 pandemic.

**Table 5:** Physicians' perception of the various facets of COVID-19 COVID-19: coronavirus disease 2019 Data is expressed in frequency (percentages).

Questions	Yes	No	Maybe
Do you think using complex personal protective equipment (PPE) increases the risk of self-contamination among healthcare workers?	87 (25.1%)	94 (27.2%)	165 (47.7%)
Do you think patients with mild respiratory symptoms from COVID-19 should be isolated at home while avoiding close contact with family members?	301 (87%)	28 (8.1%)	17 (4.9%)
Do you think healthcare workers are subjected to significant stress during the COVID-19 pandemic?	335 (96.8%)	3 (0.9%)	08 (2.3%)
If you exhibit any respiratory symptoms, would you continue providing care to the patients?	31 (9.0%)	250 (72.3%)	65 (18.8%)

With regards to the provision of PPE, our study found that the provision of disposable gloves (p-value = 0.017) and the provision of isolation gown (p-value = 0.012) are significantly associated with the type of hospital in question (government or private). Concerning the provision of training, an introduction to the methods of nasopharyngeal/oropharyngeal swab sampling was found to be significantly associated with the type of hospital (p-value = 0.001), as demonstrated by Table 6.

**Table 6:** Comparison between the provision of PPE across public and private sector hospitals PPE: personal protective equipment; COVID-19: coronavirus disease 2019 Data is expressed in frequency (percentages). *Chi-square test

Questions	Private	Public	p-value*
Provision of PPE			
Have you been provided with disposable gloves in your department?			0.017
No	26 (60.5%)	17 (39.5%)	
Yes	125 (41.3%)	178 (58.7%)	
Have you been provided with an isolation gown in your department?			0.012
No	77 (51.3%)	73 (48.7%)	
Yes	74 (37.8%)	122 (62.2%)	
Have you been provided with eye-protective goggles in your department?			0.461
No	113 (44.8%)	139 (55.2%)	
Yes	38 (40.4%)	56 (59.6%)	
Have you been provided with alcohol-based sanitizers in your department?			0.077
No	22 (33.8%)	43 (66.2%)	
Yes	129 (45.9%)	152 (54.1%)	
Provision of training			
Have you been given training regarding protective measures to be taken while encountering a patient suspected of having COVID-19?			0.532
No	95 (42.4%)	129 (57.6%)	
Yes	56 (45.9%)	66 (54.1%)	
Have you been introduced to the methods of nasopharyngeal/oropharyngeal swab sampling?			< 0.001
No	80 (32.5%)	166 (67.5%)	
Yes	71 (71%)	29 (29%)	
Have you been given training on how to use and dispose of personal protective equipment (PPE)?			0.642
No	101 (42.8%)	135 (57.2%)	
Yes	50 (45.5%)	60 (54.5%)	

Regarding the perception of doctors, responses to the question, ‘patients with mild respiratory symptoms from COVID-19 should be isolated while avoiding close contact with family members’ were significantly associated with the level of qualification (p-value = 0.013). The remaining insignificant associations are delineated in Table 7.

**Table 7:** Perception of doctors at varying designations PPE: personal protective equipment; COVID-19: coronavirus disease 2019 *Chi-square test Data is expressed in frequency (percentages). The frequencies (percentages) of doctors who responded Yes to the above-mentioned questions were tabulated.

Questions	House officer	Medical officer	Postgraduate trainee	Consultant	p-value*
Do you think using complex personal protective equipment (PPE) increases the risk of self-contamination among healthcare workers? (Yes)	29 (34.1%)	10 (11.8%)	35 (41.2%)	11 (12.9%)	0.367
Do you think patients with mild respiratory symptoms from COVID-19 should be isolated at home while avoiding close contact with family members? (Yes)	96 (33.6%)	35 (12.2%)	127 (44.4%)	28 (9.8%)	0.013
Do you think healthcare workers are subjected to significant stress during COVID-19 pandemic? (Yes)	114 (35.7%)	39 (12.2%)	135 (42.3%)	31 (9.7%)	0.152
If you exhibit any respiratory symptoms, would you continue providing care to the patients? (Yes)	7 (24.1%)	05 (17.2%)	15 (51.7%)	02 (6.9%)	0.473

## Discussion

As a developing country, with an inadequate healthcare system, Pakistan is facing a lot of challenges amid the prevailing scenario. The overarching aim of this study was to assess the readiness of the doctors in various hospitals across Pakistan to combat the pandemic with the scarce facilities and training opportunities available. In our study population, 28.9% of healthcare workers claimed to be exposed to COVID-19 suspected patients almost daily, and 50.9% of the respondents claimed occasional exposure. Of the population, 46.5% was working for more than five days a week. These percentages reveal that a large proportion of doctors working in different health care facilities across Pakistan is predisposed to a constantly increasing number of COVID-19 suspected patients.

The healthcare professionals are jeopardized to the risk of infection due to several factors and must be provided with adequate personal protective equipment (PPE), including masks, gloves, goggles, gowns, hand sanitizers, and cleaning supplies [7-8]. A cross-sectional study including doctors in the United States and Pakistan demonstrated that 50.6% of the Pakistani doctors had been compelled to work without PPE as compared to merely 7.1% of the doctors in the US [9]. In our survey, 72.8% and 43.4% of respondents professed to have no access to eye protective equipment and gowns, respectively. Responses to the questions regarding the provision of PPE were compared among the subjects working in public and private health sectors. The results show that 60.5% of respondents from the private hospitals were not provided with adequate disposable gloves (p-value = 0.017), indicating that these variables are related. Similarly, 51.3 % of respondents from private hospitals were not provided with isolation gowns (p-value = 0.012). These results might suggest that the difference between the provision of PPE among the public and private sectors of healthcare facilities is due to the government attempting to make sure its ample supply for the healthcare workers working in the public sector. It might also be due to increased financial strain due to which private hospitals in developing countries have difficulty in providing their healthcare workers with appropriate facilities. To ensure a better outcome during the pandemic, there is an unmet need for a sound collaboration between the private sector hospitals and government health care policymakers [8]. Many health care workers have been reported to die, with hundreds being infected with COVID-19 in Pakistan [5,10]. The satisfactory provision of PPE can, therefore, protect the healthcare workers by lessening the transmission rate, thereby reducing the stress on the vulnerable healthcare system.

An essential component of the readiness of the doctors working in various departments during the current situation is their level of training regarding protective measures to be taken while contracting a speculated patient and treating them simultaneously, the use and disposal of PPE, and the techniques of nasopharyngeal/oropharyngeal swab sampling. Our survey demonstrated that only 35.3% of the respondents were being trained properly regarding the protective measures to be taken while encountering a patient suspected of having COVID-19, 28.95% being introduced to the methods of nasopharyngeal/oropharyngeal swab sampling, and 31.8% being given training on how to use and dispose off PPE. These results challenge the inclination of our front-line healthcare workers to combat the prevailing circumstance. Another study showed that about 76% of the front-line healthcare workers had some level of confidence in isolating a suspected case while 72% of respondents of the study felt somewhat confident about what and how to use PPE [11]. The disparity in these percentages signifies that the level of training of doctors in our hospitals is notably unsatisfactory regarding PPE usage. We also noted a significant difference regarding the training of nasopharyngeal/oropharyngeal swab sampling between doctors working public and private setups. Amongst the respondents, 76% serving in private hospitals were introduced to the methods of nasopharyngeal swab sampling while only 29% of doctors working in public hospitals said they knew the method, with a significant p-value of < 0.001, indicating that these variables were related. However, there was no statistically significant difference between the level of training regarding the use of PPE and precautionary measures to take while treating a COVID-19 suspected patient among the doctors working in the public and private sectors. Our healthcare organizations must ensure the provision of formal training and guidance to our doctors in order to make our healthcare system more qualified in contending fatal infectious diseases.

The perception level of the doctors regarding COVID-19 might be a contributing factor for adequate preparedness. Although the use of more complex PPE may increase the risk of self-contamination, only 25.1% of respondents agreed to this and 47.7% were not sure about it [12]. The results were not significantly associated with doctors working at various designations. Patients with mild COVID-19 symptoms can be isolated and managed at home while avoiding close contact with family members [13]. Of the doctors, 86.9% agreed to this notion, with the response being statistically significant (p-value = 0.013).

With the emergence of the COVID-19 pandemic, hospitals are compelled to propose new policies and practices among which are the introduction of proper triage systems to receive and screen suspected patients, the availability of portable imaging equipment, and sound communication among the hospital management, departments, and front-line healthcare workers [8,12]. Forty-three point four percent (43.4%) of individuals responded that they had no triage system and portable imaging equipment in their respective departments. A triage plan enables us to designate our healthcare resources more effectively and prevents its unwarranted use [14]. Furthermore, valuable communication in times of crisis plays a pivotal part in improving the fecundity of a healthcare system, including two-way communication between front-line providers and the leadership [15]. Amongst the respondents, 53.5% claimed sound collaboration between healthcare representatives of the respective departments and hospital management. However, a large percentage responded to the opposite. Our survey also demonstrated that 86.7% of the doctors notified their hospital’s infection prevention team or local health department when a COVID-19 suspected patient was being investigated, alluding to a good reporting practice.

Front-line healthcare workers are subjected to a significant amount of strain amid the pandemic, which can be contributed to numerous factors. In our survey, 96.9% of the doctors thought that healthcare workers were subjected to significant stress during the COVID-19 pandemic. One of the factors contributing to the mental stressors among the doctors can be the fear of getting infected themselves, and 94.6% of respondents were self-monitoring themselves for signs of illness. Another major contributing factor to the level of anxiety can be the fear of transmitting the infection to family members, and 98.3% of doctors responded that they were concerned about the possibility. A study conducted in China assessed the degree of mental health symptoms among the front-line workers and reported that more than 70% of participants had psychological symptoms [16]. Providing mental healthcare is indispensable in helping our doctors overcome the psychological strains exerted by the pandemic. To monitor their mental health, physicians must be provided with adequate mental health resources. However, in our study, 85.3% of the doctors said they were not being provided with mental health and counseling resources in their departments. This might be one of the factors affecting the readiness of our doctors in combating the COVID-19 pandemic unfavorably.

Nevertheless, using an online survey distributed via various social platforms remains a limitation of our study. This can potentially explain the unequal representation of doctors in various departments across the country.

## Conclusions

In Pakistan, doctors working on the frontline of the public sector amidst the COVID-19 pandemic have a better provision of PPE. Special attention should be given to private-sector doctors pertaining to the factors posing difficulties to the proper supply, demand, and use of PPE. It is also imperative to provide adequate training to the frontline workforce, along with education to improve their perceptions about the disease, training, and patient care. A large proportion of the respondents believed that doctors are under significant mental stress and the issue must be addressed appropriately by facilitating good communication and collaboration among healthcare workers, respective departments, and local health authorities. Moreover, mental health counseling resources must also be provided to doctors.

## References

[1] Guo YR, Cao QD, Hong ZS (2020). The origin, transmission and clinical therapies on coronavirus disease 2019 (COVID-19) outbreak - an update on the status. Mil Med Res.

[2] Shah K, Chaudhari G, Kamrai D, Lail A, Patel RS (2020). How essential is to focus on physician's health and burnout in coronavirus (COVID-19) pandemic?. Cureus.

[3] Sohrabi C, Alsafi Z, O'Neill N (2020). World Health Organization declares global emergency: a review of the 2019 novel coronavirus (COVID-19). Int J Surg.

[4] Singhal T (2020). A review of coronavirus disease-2019 (COVID-19). Ind J Pediatr.

[5] Saqlain M, Munir MM, Ahmed A, Tahir AH, Kamran S (2020). Is Pakistan prepared to tackle the coronavirus epidemic?. Drugs Ther Perspect.

[6] Chang D, Xu H, Rebaza A, Sharma L, Dela Cruz CS (2020). Protecting health-care workers from subclinical coronavirus infection. Lancet Resp Med.

[7] (2020). WHO. Coronavirus disease (COVID-19) outbreak: rights, roles and responsibilities of health workers, including key considerations for occupational safety and health. https://www.who.int/docs/default-source/coronaviruse/who-rights-roles-respon-hw-covid-19.pdf?sfvrsn=bcabd401_0.

[8] Shadmi E, Chen Y, Dourado I (2020). Health equity and COVID-19: global perspectives. Int J Equity Health.

[9] (2020). CDC. Healthcare professional preparedness checklist for transport and arrival of patients with confirmed or possible COVID-19. https://www.cdc.gov/coronavirus/2019-ncov/hcp/hcp-personnel-checklist.html.

[10] Javed B, Sarwer A, Soto EB, Mashwani ZUR (2020). Is Pakistan's response to coronavirus (SARS-CoV-2) adequate to prevent an outbreak?. Front Med.

[11] Prescott K, Baxter E, Lynch C, Jassal S, Bashir A, Gray J (2020). COVID-19: how prepared are front-line healthcare workers in England?. J Hosp Infect.

[12] Herron JBT, Hay-David AGC, Gilliam AD, Brennan PA (2020). Personal protective equipment and Covid 19- a risk to healthcare staff?. Br J Oral Maxillofac Surg.

[13] Chughtai AA, Khan W (2020). Use of personal protective equipment to protect against respiratory infections in Pakistan: a systematic review. J Infect Public Health.

[14] Lai J, Ma S, Wang Y (2020). Factors associated with mental health outcomes among health care workers exposed to coronavirus disease 2019. JAMA Netw Open.

[15] Maves RC, Downar J, Dichter JR (2020). Triage of scarce critical care resources in COVID-19 an implementation guide for regional allocation. Chest.

[16] Garg M, Wray CM (2020). Hospital medicine management in the time of COVID-19: preparing for a sprint and a marathon. J Hosp Med.

